# HIV Encephalopathy Mimicking Acute Demyelinating Processes

**DOI:** 10.7759/cureus.18494

**Published:** 2021-10-05

**Authors:** Wasey Ali Yadullahi Mir, Dhan B Shrestha, Francesco Fiumara, Sunita Mohapatra, Thomas Sullivan, Anurag Adhikari, Larissa Verda

**Affiliations:** 1 Internal Medicine, Mount Sinai Hospital, Chicago, USA; 2 Infectious Diseases, Mount Sinai Hospital, Chicago, USA; 3 Radiology, Mount Sinai Hospital, Chicago, USA; 4 Emergency Medicine, Shankarapur Hospital, Kathmandu, NPL

**Keywords:** acquired immunodeficiency syndrome, human immunodeficiency virus, multiple sclerosis, leukoencephalopathy, antiretroviral therapy

## Abstract

Human immunodeficiency virus (HIV) encephalopathy lies in the severe spectrum of HIV-associated neurological disorder (HAND) and ranges from asymptomatic condition to minor neurological features to severe dementia. Cerebrospinal fluid (CSF) analysis helps to rule out the presence of other opportunistic infections. Neuroimaging helps establish the diagnosis.

We report a case of a 39-year-old African American female who presented with signs and symptoms suggestive of acute multiple sclerosis (MS) flares in the setting of advanced acute immunodeficiency syndrome (AIDS) encephalopathy. She presented with bilateral lower extremity muscle weakness and pain with apparent cognitive decline. Notable laboratory findings included leukopenia with normal neutrophils and positive serology for HIV-1. The MRI showed mild post-contrast enhancement suggestive of demyelinating disease, favoring MS over progressive multifocal leukoencephalopathy (PML). Cerebrospinal fluid analysis was significant for positive oligoclonal bands and negative serology. She was started on antiretroviral therapy (ART) for AIDS while holding steroids due to the possibility of worsening AIDS. After treatment for HIV, she showed immunologic and functional status improvement. HIV encephalopathy must be diagnosed by ruling out other similar presenting neurological illnesses for tactful patient management.

## Introduction

Acquired immunodeficiency syndrome (AIDS) is a state of decreased immune function, whereas multiple sclerosis (MS) is the state of immune-mediated destruction of the nervous system. In an immunocompromised state, it is very unlikely for the body to launch a significant auto-immune attack. Studies have shown that having HIV significantly decreases the risk of developing MS [1]. Many treatments for MS are immunosuppressive agents, which are theoretically the same immune functions that are suppressed by an AIDS infection. On the other hand, treatment for AIDS reverses the suppressed immune status and cluster of differentiation 4 (CD4)+T cells. Studies show that those mediators of immune function are associated with the autoimmune demyelinating destruction seen in MS [2]. Therefore, concurrent symptomatology of AIDS and MS is highly improbable and poses a diagnostic and therapeutic challenge that requires intensive workup and thorough investigation of other possibilities.

## Case presentation

A 39-year-old African American female with a past medical history of non-toxic goiter and reflux esophagitis presented to our hospital for bilateral lower extremity muscle weakness and pain for two to three weeks. Her inability to ambulate had resulted in two falls, and she reported a 30-pound weight loss over three months attributed to poor appetite. On arrival, she had a fever of 100.7 ^o^F but was otherwise hemodynamically stable. She had a cachectic appearance with an apparent cognitive decline, notably slow thought process and reaction time. Oral thrush was present, no weakness in bilateral upper extremities, tender to touch bilateral lower extremities, weakness was predominant on proximal lower extremity (2/5) muscles than distal (4/5), normal sensation, and flexor plantar response. Reflexes were normal throughout, with somewhat brisk reflex on the right upper extremity. She also reported urinary retention but good bowel control.

**Figure 1:**
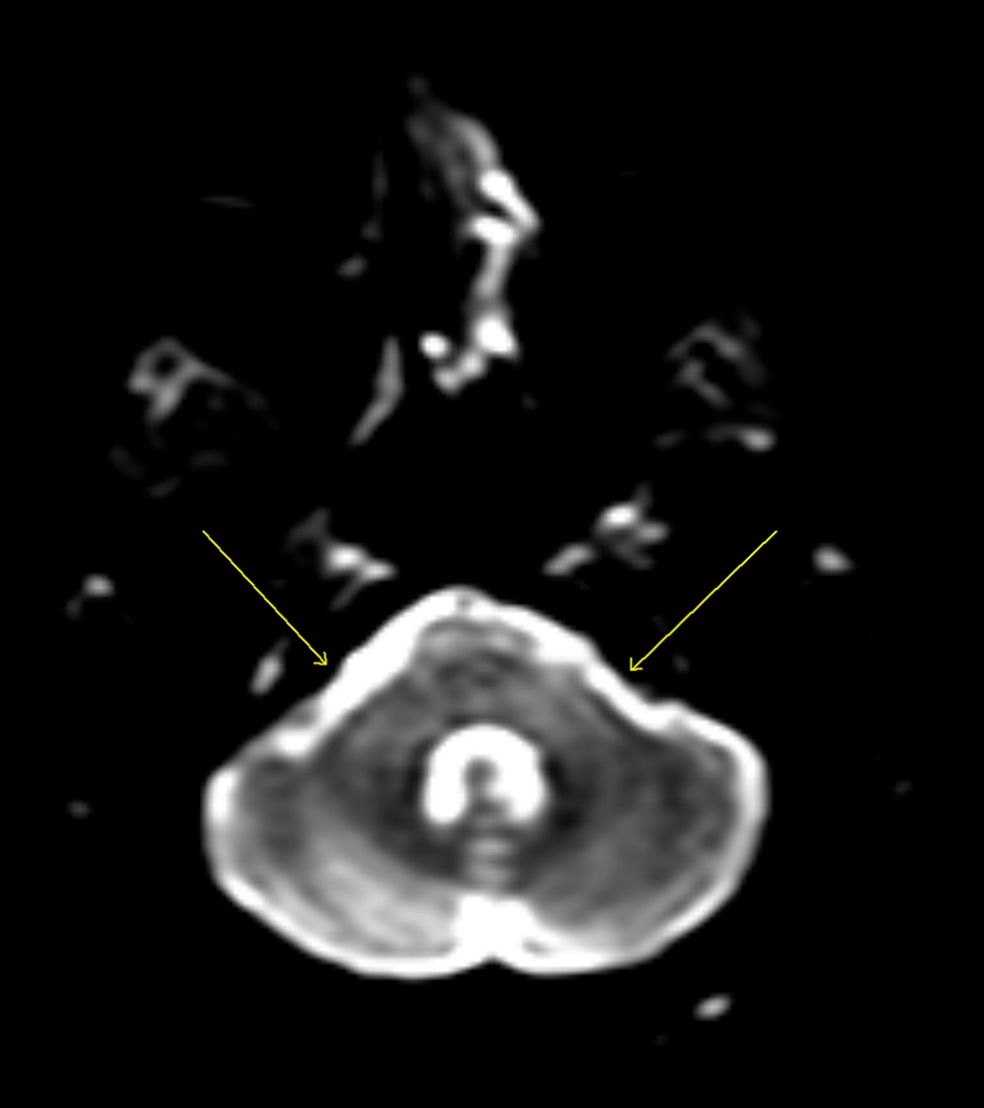
Diffusion-weighted MRI when the patient presented. Arrows show both brachium (left>right) of pons with extensive hyperintense T2-signal abnormality (also present over pericallosal and periventricular white matter suggesting demyelination).

**Figure 2:**
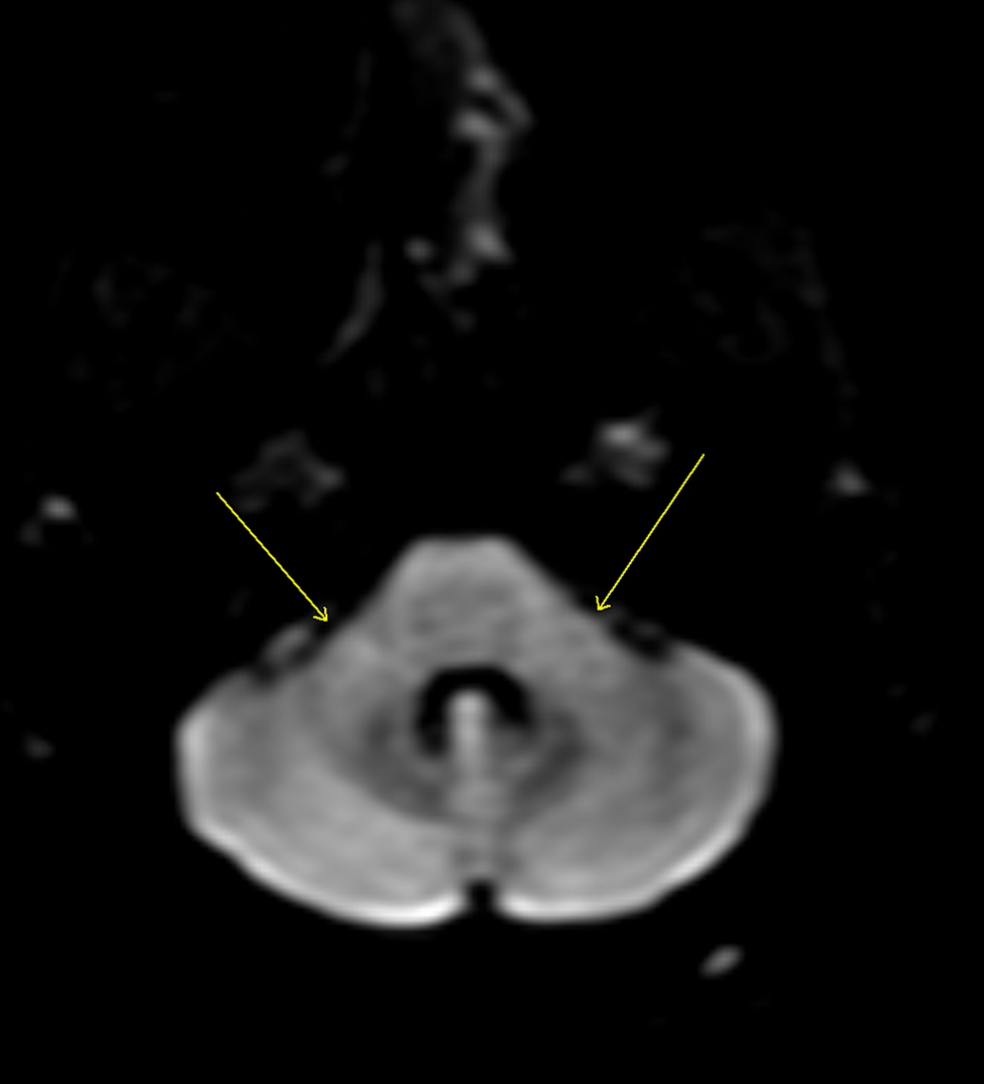
Post-contrast diffusion-weighted MRI when the patient presented. The arrows show brachium pontis.

**Figure 3:**
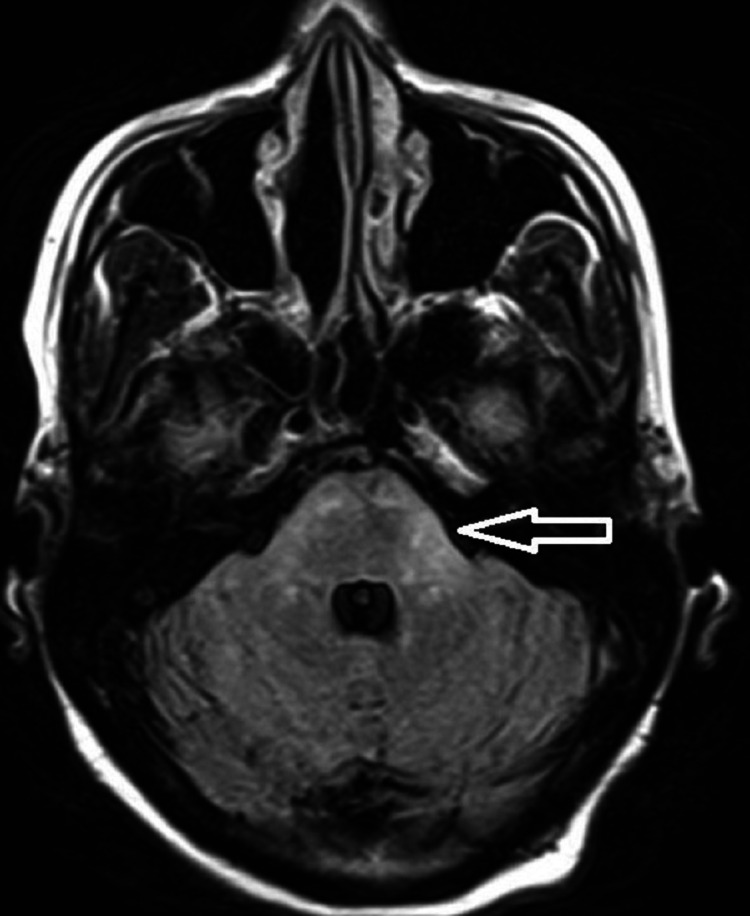
FLAIR image with an arrow showing hyperintense signal abnormality over left brachium pontis FLAIR: Fluid-attenuated inversion recovery

**Figure 4:**
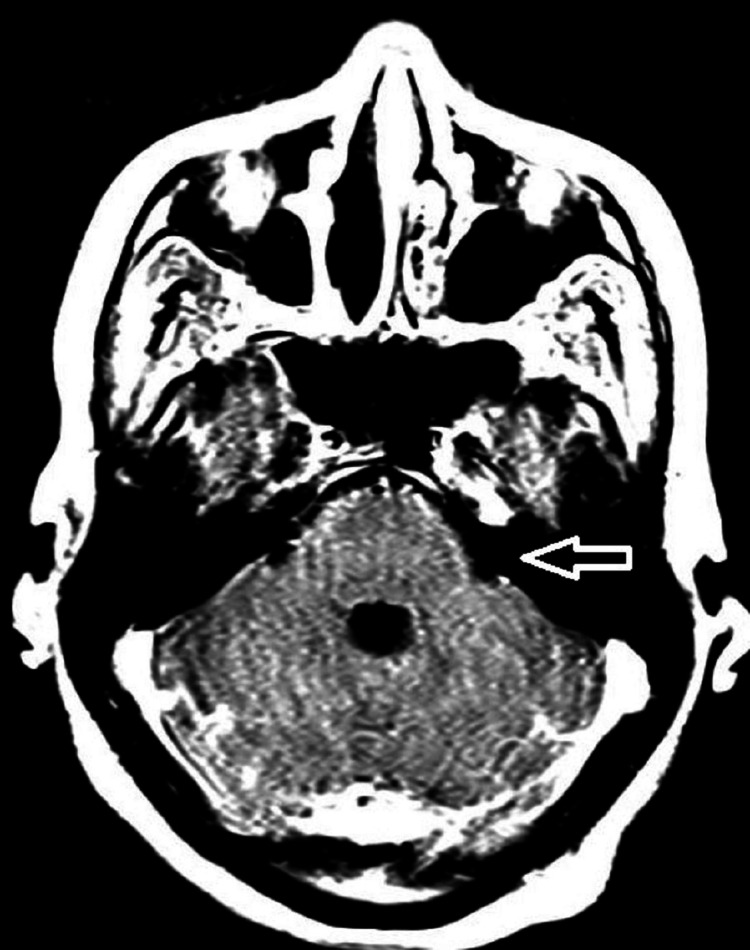
Post-contrast FLAIR imaging with the arrow showing equivocal contrast with mild enhancement. FLAIR: Fluid-attenuated inversion recovery

The laboratory findings on arrival were significant for leukopenia with normal neutrophils count, creatine kinase (CK) of 5171 international unit/liter (IU/L), erythrocyte sedimentation rate of 62 mm/hr, and positive serology for HIV-1. Her CD4 count was 22 cells/mm^3^ and her viral load was 172,000 copies/ml. Magnetic resonance imaging (MRI) with contrast was significant for extensive abnormal hyperintense T2-weighted-fluid-attenuated inversion recovery (T2-FLAIR) signals in the pericallosal and periventricular white matter, brainstem, and bilateral brachium pontis with equivocal mild post-contrast enhancement suggestive of demyelinating disease (Figures 1-4) favoring MS over progressive multifocal leukoencephalopathy (PML). Cerebrospinal fluid (CSF) analysis showed lymphocyte-predominant raised white blood cell (WBC) count (3000/mm^3^) with normal glucose and protein, high immunoglobulin G (IgG) index and synthesis rate, and three well-defined oligoclonal bands.

Acid-fast bacilli (AFB), mycobacterium, fungal, and mycobacterium avium complex (MAC) cultures, polymerase chain reaction (PCR) of John Cunningham (JC) virus and cytomegalovirus, cryptococcus antigen, Epstein-Barr virus (EBV), and Herpes 1 and 2 antibodies in CSF and rapid plasma reagin (RPR) were all negative. The MRI of the lumbar, thoracic, and cervical spine was negative for any demyelinating lesions. The CK normalized and autoimmune workup was negative. No signs of optic neuritis or cytomegalovirus (CMV) retinitis were found on an eye exam.

**Figure 5:**
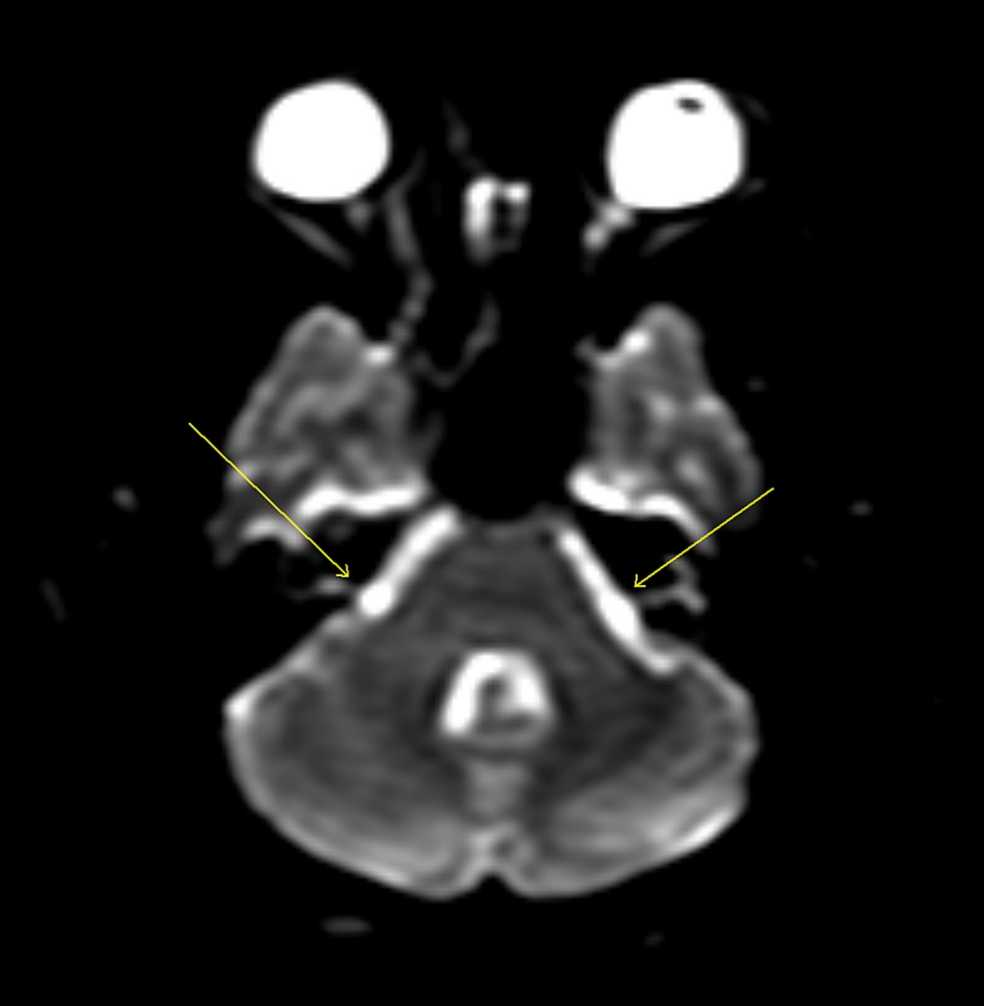
Diffusion-weighted MRI. The arrows show marked improvement in the brachium pontis lesion.

**Figure 6:**
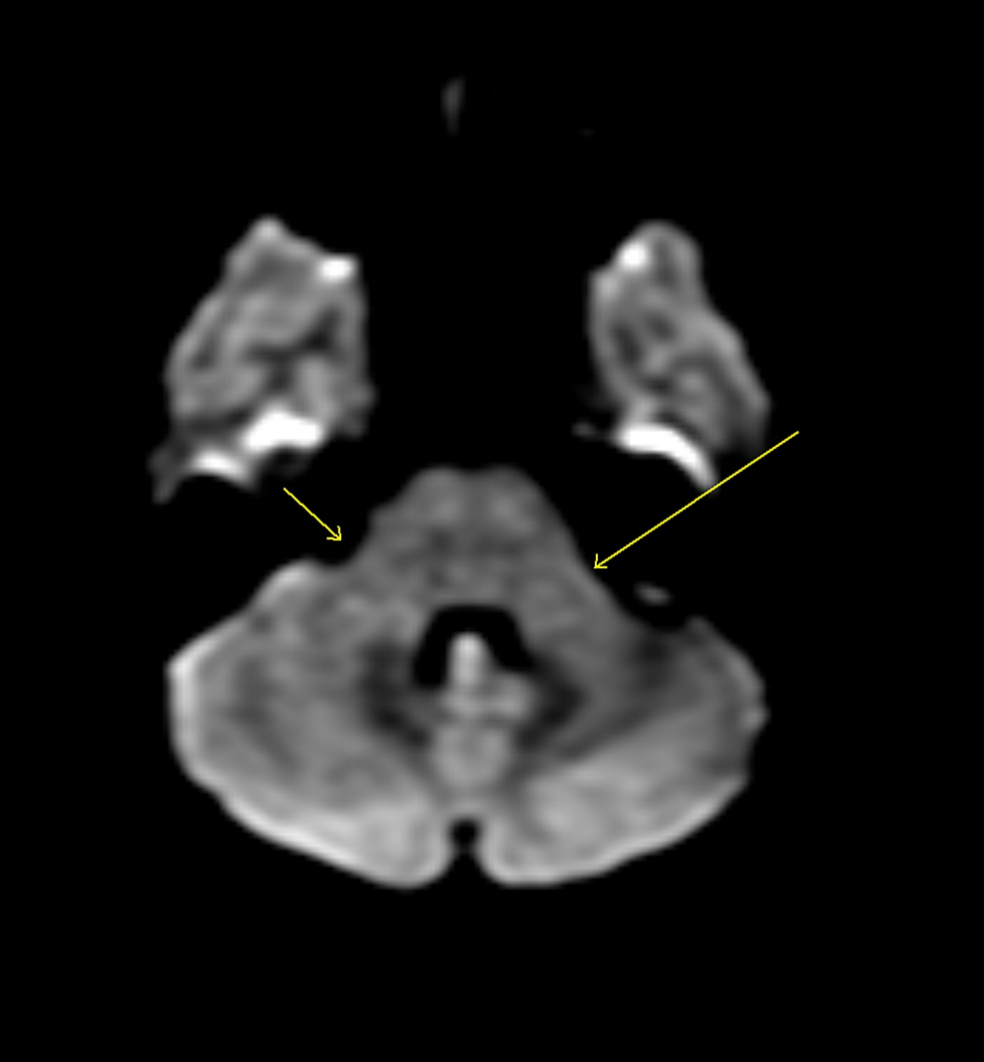
Diffusion-weighted MRI, post-contrast phase. The arrows show marked improvement in the brachium pontis lesion.

**Figure 7:**
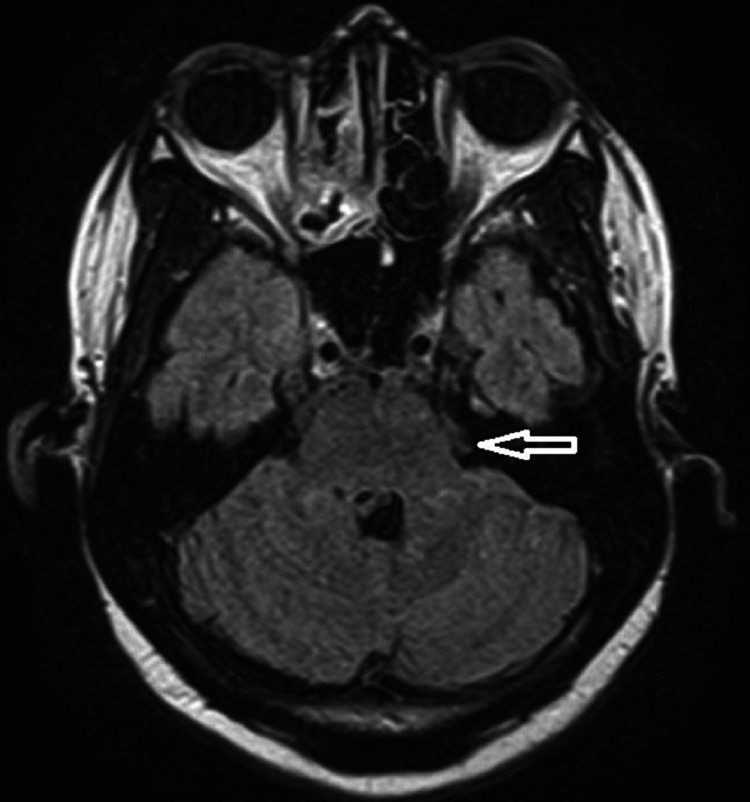
FLAIR imaging. The arrow shows the resolution of the hyperintense lesion in the left brachium pontis. FLAIR: Fluid-attenuated inversion recovery

**Figure 8:**
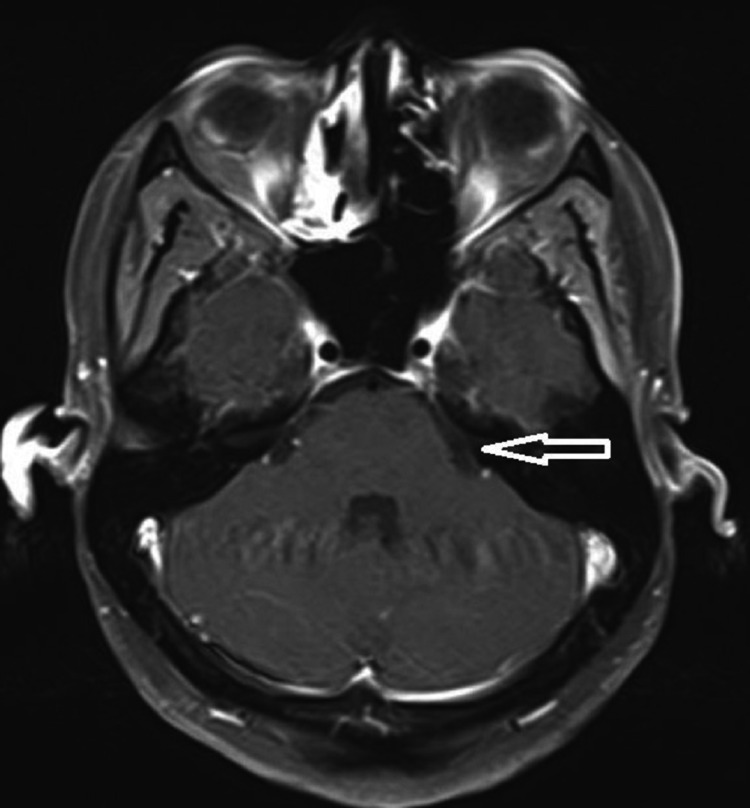
FLAIR imaging post-enhancement. The arrow points at the resolution of the prior enhancement. FLAIR: Fluid-attenuated inversion recovery

She was started on antiretroviral (ART), which included tenofovir and emtricitabine, along with fluconazole for thrush and dapsone prophylaxis for pneumocystis. The ART was started in 2019 with 100% adherence to the regimen. Steroids were held due to the risk of worsening of AIDS. After a few days of therapy, her cognitive status and weakness showed mild improvement, and she was discharged to an acute rehabilitation center. Following one month of rehabilitation and an ART, her cognitive level and strength showed significant improvement. She could walk unassisted. Her CD4 count rose to 545 cells/mm^3^, and viral load decreased to 181 copies/ml. Currently, she is ambulatory and can perform activities of daily living independently. Neurologically, her memory and recall functions have improved. A follow-up MRI in June 2021 showed improvement of previous lesions and no new lesions (Figures 5-8).

## Discussion

Our patient is an extremely rare case that demonstrates acute HIV encephalopathy due to a new AIDS diagnosis. There has been one published case report of a patient with MS and HIV treated with antiretroviral drugs [3]. However, the final diagnosis proved difficult because her presenting findings were reported to be consistent with AIDS, such as oral thrush and a CD4 count of 22 cells/mm^3^.Still, her neurologic symptoms, cerebrospinal fluid findings, and her demographic, age, and female sex strongly suggested multiple sclerosis as well. Furthermore, her clinical presentation had overlapping neurological symptoms, which prompted the consideration to rule out progressive multifocal leukoencephalopathy.

It has been well studied that both CD4+ and CD8+ cells are active in acute lesions due to multiple sclerosis. However, the frequency of CD8+ cells is more in chronic cases of multiple sclerosis, whereas CD4+ cells are responsible for the majority of an acute flare and the extent of the lesion [2]. Furthermore, treatment targeting T cells is a well-documented model of effective pharmacotherapy for treating multiple sclerosis.

HIV encephalopathy, the final diagnosis

HIV encephalopathy usually occurs as a result of increased viral load due to untreated AIDS in a patient. This is what happened with our patient. However, unlike in our patient, the early course of the disease is usually asymptomatic. When symptoms are present, it may impact anywhere in the central nervous system (CNS), but most frequently affects the subcortical tissue first [4,5]. Our patient had MRI findings consistent with lesions involving the periventricular white matter, brainstem, and bilateral brachium pontis, consistent with the broad scope of inclusion for this disease course. Moreover, HIV encephalopathy can demonstrate incomplete symmetry and is expected to affect the periventricular white matter [6]. This manifested not only on the MRI findings but also on the clinical picture of the patient, presenting with bilateral lower extremity weakness and difficulty ambulating.

The patient was compliant with her medications, and after initiating antiretroviral therapy, her CD4 count increased from 22 cells/mm^3^ to 545 cells/mm^3,^ and her viral load decreased from 172,000 copies/ml to 181 copies/ml. In addition, the patient's symptoms were much less severe after restoring reliable immune function and adhering to her antiretroviral medication regimen. After the treatment of her AIDS, restoration of CD4 counts, and with the help of therapy, she was eventually able to walk unassisted. This is a crucial finding as this treatment often results in near-complete resolution in patients with acute HIV encephalopathy with strict adherence to antiretroviral therapy and restoration of the CD4 count.

This is a rare case of HIV encephalopathy due to multiple factors, including the patient's age, the new diagnosis of HIV and AIDS, and the patient's clinical presentation. Most frequently, HIV encephalopathy occurs as a late complication of the viral infection [5]. This is a young patient who must have had an underlying infection that went untreated for a prolonged period. The symptoms manifested abruptly due to a very low CD4 count and high viral load due to untreated AIDS. However, when HIV encephalopathy occurs acutely, it is often associated with neurocognitive delay or dysfunction symptoms. Our patient did not experience any confusion, altered sensorium, or dementia-related symptoms. Instead, her presentation was entirely motor in nature, prompting the unusual presentation that was considered against other diagnoses.

Multiple sclerosis, the differential diagnosis

The patient is a 39-year-old woman who is within the typical range of autoimmune diseases and presented with difficulty walking due to acute lower extremity weakness. She also presented with oligoclonal bands on CSF analysis, which are highly sensitive, but not specific to MS. Furthermore, the MRI findings demonstrating hyperintense T2-FLAIR lesions in the periventricular area are consistent with MS. However, what eventually helped rule out the diagnosis of MS was the patient's response to treatment. Initiating antiretroviral therapy significantly improved the patient's CD4 count, and more importantly clinical picture. An acute flare of MS is treated most commonly with steroids. Unfortunately, corticosteroids precipitate an immunosuppressed state, which was a significant concern for our patient with an active HIV infection. For this reason, the steroids were put on hold until antiretroviral therapy proved successful in improving her CD4 counts and decreasing her viral load.

It has been known that both CD4+ and CD8+ cells are active in acute lesions due to multiple sclerosis. The treatment targeting T cells is a well-documented method of effective pharmacotherapy for treating MS. This helps to explain the reasoning as to why the patient had the diagnosis of acute HIV encephalopathy as compared to MS. A patient with such a low functioning immune system is highly unlikely to develop inflammatory-mediated destruction of the central nervous system. This is mainly because the primary mode of damage is through the CD4 cells which are low in the patient. Moreover, the patient did not experience a subsequent acute multiple sclerosis flare after restoring the function of CD4 cells from 22 cells/mm^3^ to 545 cells/mm^3^; rather, the symptoms subsided with the treatment of the HIV infection alone. Additionally, it has been theorized that depleting CD4 cells in HIV is protective against an exacerbation of MS [7].

Rarely, patients with AIDS who have a very low CD4 count can present with an acute multiple sclerosis flare. However, a sizeable comparative cohort study including 21207 HIV-positive patients found the rate ratio of developing MS in people with HIV to be 0.38 (95% CI 0.15 to 0.79) [1,3,8]. The symptoms of an acute multiple sclerosis flare could also be due to the overactivity of CD8 cells, the pathology of HIV/AIDS that destroys CD4 cells.

Progressive multifocal leukoencephalopathy, the differential diagnosis

While the MRI findings can be consistent with demyelinating foci in the subcortical and periventricular areas of the brain, it is much less common to find symmetry in lesions due to progressive multifocal leukoencephalopathy [6]. While this may seem like a clear distinction, it is not always clear on MRI, nor is it diagnostic. The PML due to the JC virus may present similar clinical findings to both MS and acute HIV encephalopathy. Patients can often present with difficulty ambulating, weakness in their lower extremities, and ataxia. The PML can be diagnosed with CSF analysis for the presence of the JC virus. However, on analysis of the patient's CSF, there was no evidence of the JC virus. Due to higher clinical suspicion for other acute processes in conjunction with the CSF findings, PML was effectively ruled out.

## Conclusions

The coexistence of demyelinating neurological disorders and HIV is very rare. HIV infection is associated with a significantly decreased risk of developing MS. It has been reported that in patients with MS who acquired HIV, their symptoms resolve completely, as immunosuppression induced by chronic HIV infection and antiretroviral medications is a protective association for MS. In our case, when PML was ruled out, imaging and lab findings suggested multiple sclerosis. With no prior diagnosis or symptomatology of MS, and a significant response to ART raises the question of atypical presentation of demyelination and presence of an oligoclonal band in HIV-induced encephalopathy.

## References

[1] Gold J, Goldacre R, Maruszak H, Giovannoni G, Yeates D, Goldacre M (2015). HIV and lower risk of multiple sclerosis: beginning to unravel a mystery using a record-linked database study. J Neurol Neurosurg Psychiatry.

[2] Chitnis T (2007). The role of CD4 T cells in the pathogenesis of multiple sclerosis. Int Rev Neurobiol.

[3] Maruszak H, Brew BJ, Giovannoni G, Gold J (2011). Could antiretroviral drugs be effective in multiple sclerosis? A case report. Eur J Neurol.

[4] Valcour V, Sithinamsuwan P, Letendre S, Ances B (2011). Pathogenesis of HIV in the central nervous system. Curr HIV/AIDS Rep.

[5] Daliparty VM, Balasubramanya R (2021). HIV encephalitis. https://pubmed.ncbi.nlm.nih.gov/32310354/.

[6] Haziot ME, Barbosa Junior SP, Vidal JE, de Oliveira FT, de Oliveira AC (2015). Neuroimaging of HIV-associated neurocognitive disorders. Dement Neuropsychol.

[7] Skarlis C, Gontika M, Katsavos S, Velonakis G, Toulas P, Anagnostouli M (2017). Multiple sclerosis and subsequent human immunodeficiency virus infection: a case with the rare comorbidity, focus on novel treatment issues and review of the literature. In Vivo.

[8] Berger JR, Sheremata WA, Resnick L, Atherton S, Fletcher MA, Norenberg M (1989). Multiple sclerosis-like illness occurring with human immunodeficiency virus infection. Neurology.

